# Changing Feeding Levels Reveal Plasticity in Elasmobranch Life History Strategies

**DOI:** 10.1111/ele.70201

**Published:** 2025-09-03

**Authors:** Sol Lucas, Per Berggren, Ellen Barrowclift, Isabel M. Smallegange

**Affiliations:** ^1^ School of Natural and Environmental Sciences Newcastle University Newcastle upon Tyne UK; ^2^ Sussex Inshore Fisheries and Conservation Authority 12A Riverside Business Centre Shoreham UK; ^3^ Marine Research and Conservation Foundation (MARECO), Emble Farm Lydeard St Lawrence UK

**Keywords:** conservation biology, demographic resilience, elasmobranch, generation turnover, individual energy budgets, integral projection models, life history strategies, life history traits, population performance, reproductive output

## Abstract

Life history strategies are shaped by phylogeny, environmental conditions and individual energy budgets, and have implications for conservation biology. We summarised life history traits of 151 elasmobranch species into life history strategies for two contrasting feeding levels, representing two different environments, in a principal components analysis. Two axes, reproductive output and generation turnover, structure elasmobranch life history strategies. Species' positions in this life history space were not fixed but shifted to higher reproductive output when feeding level increased. We also found that both axes predicted population performance, but that population growth rate does not necessarily inform on a species' demographic resilience. Finally, neither axis predicted IUCN conservation status. Our analyses reveal plasticity in species life history strategies and warn against extrapolating the life history strategy framework from one environment to another when predicting a species' response to (climate) change, perturbations, and (over)exploitation.

## Graphical abstract

We analysed life history traits of 151 elasmobranch species for two contrasting feeding levels in a principal components analysis. Two axes, reproductive output and generation turnover, structure elasmobranch life history strategies. Wefound that species' positions in this life history space are dynamic, shifting towards higher reproductive output as feeding levels increase.

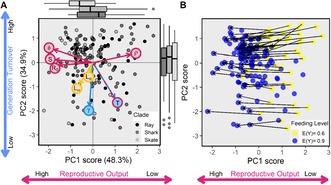

## 1 Introduction

Life history strategies of animals and plants reflect trade‐offs at the species level. For example, the fast‐slow continuum represents the trade‐off between reproduction versus survival (Stearns 1983), often irrespective of body size or phylogenetic relatedness (Williams 1966; Gadgil et al. 1970; Reznick 1983; Gaillard et al. 2016). Yet, trade‐offs also operate within individuals, such as trading off growth versus reproduction in the energy budget (Gadgil et al. 1970; Reznick 1983), as different dynamics can exist between populations or species with similar traits (Nilsen et al. 2009; Gamelon et al. 2021). Specifically, the classical association between life history strategies and population responses to environmental change can break down in variable environments when accounting for individual‐level trade‐offs and energy allocation during the development of individuals (Rademaker et al. 2024). One reason for this could be that individual‐level plasticity allows organisms to adjust energy allocation in response to environmental variability, maintaining stability under resource limitation and accelerating growth and reproduction in favourable conditions (Weidner et al. 2020).

Calls exist for more theory‐driven studies of how plasticity in development shapes life history variation (Stott et al. 2024). Additionally, we have limited understanding of how ecological and evolutionary factors, like phylogeny or habitat, shape variation in life history strategies across species (Salguero‐Gómez et al. 2016; Salguero‐Gómez 2017; Capdevila, Beger, et al. 2020). Identifying patterns and plasticity in life history strategies across the tree of life is one way to predict population growth rates and demographic resilience (combinedly referred here as ‘population performance’) (Salguero‐Gómez et al. 2016). However, this requires an in‐depth understanding of how energy budgets, phylogeny and habitat structure life history strategies across species (Capdevila, Beger, et al. 2020; Romeijn and Smallegange 2022; Stott et al. 2024). Elasmobranchs (sharks, skates and rays) encompass a vast amount of life history variation. For example, longevity can range from five (e.g., *Carcharhinus sealei* ; Ebert et al. 2021) to almost 400 years ( *Somniosus microcephalus* ; Nielsen et al. 2016). Reproduction is highly variable, including oviparity (skates and three families of shark), aplacental viviparity (in various forms; sharks and rays) and placental viviparity (some sharks) (Carrier et al. 2004; Miller et al. 2022). Elasmobranch population performance typically varies with body size, reproductive strategy and habitat (Pardo and Dulvy 2022; Barrowclift et al. 2023; Gravel et al. 2024), yet little is known about the impact of energy budgets.

Here, we investigate how individual energy budgets, phylogeny and habitat structure influence elasmobranch life history strategies across environments that differ in food availability, and if the resulting life history framework links to population performance and conservation biology (Salguero‐Gómez et al. 2016; Salguero‐Gómez 2017). Using Dynamic Energy Budget Integral Projection Models (DEB‐IPMs) for 151 elasmobranch species (Kooijman and Metz 1984; Sousa et al. 2010; Ellner et al. 2016; Smallegange et al. 2017; Smallegange and Lucas 2024), we model survival, growth and reproduction based on species‐specific energy budgets under different feeding levels (Kooijman 2001; Kooijman et al. 2008). While regression analyses test predefined trait relationships (Sol et al. 2012; Gaillard et al. 2016; Tomasek et al. 2019), principal component analysis (PCA) identifies emergent life history strategies by summarising trait variation into major axes without prior assumptions (e.g., Gaillard et al. 2016; Salguero‐Gómez et al. 2016; Paniw et al. 2018). Therefore, to identify life history strategies, we (objective 1) calculate, using the parameterised DEB‐IPMs, a set of representative life history traits based on schedules of survival, growth and reproduction for a low and a high feeding level (reflecting low and high food availability, respectively). We (objective 2) summarise these traits using a phylogenetically corrected PCA to identify major axes of elasmobranch life history strategies. We then (objective 3) assess the influence of phylogenetic ancestry (clade) and habitat (water temperature) on species' positions along these major axes, and to what extent the position of species along these axes changes with food availability (showing plasticity). Finally, (objective 4) we test whether species position along these axes predicts population growth rate, speed of recovery from perturbations (demographic resilience) and IUCN conservation status. Our approach allows us to investigate whether feeding level influences life history strategies and the resulting conservation biology of elasmobranch species.

## 2 Methods

### 2.1 Brief Description of the DEB‐IPM

**FIGURE 1:**
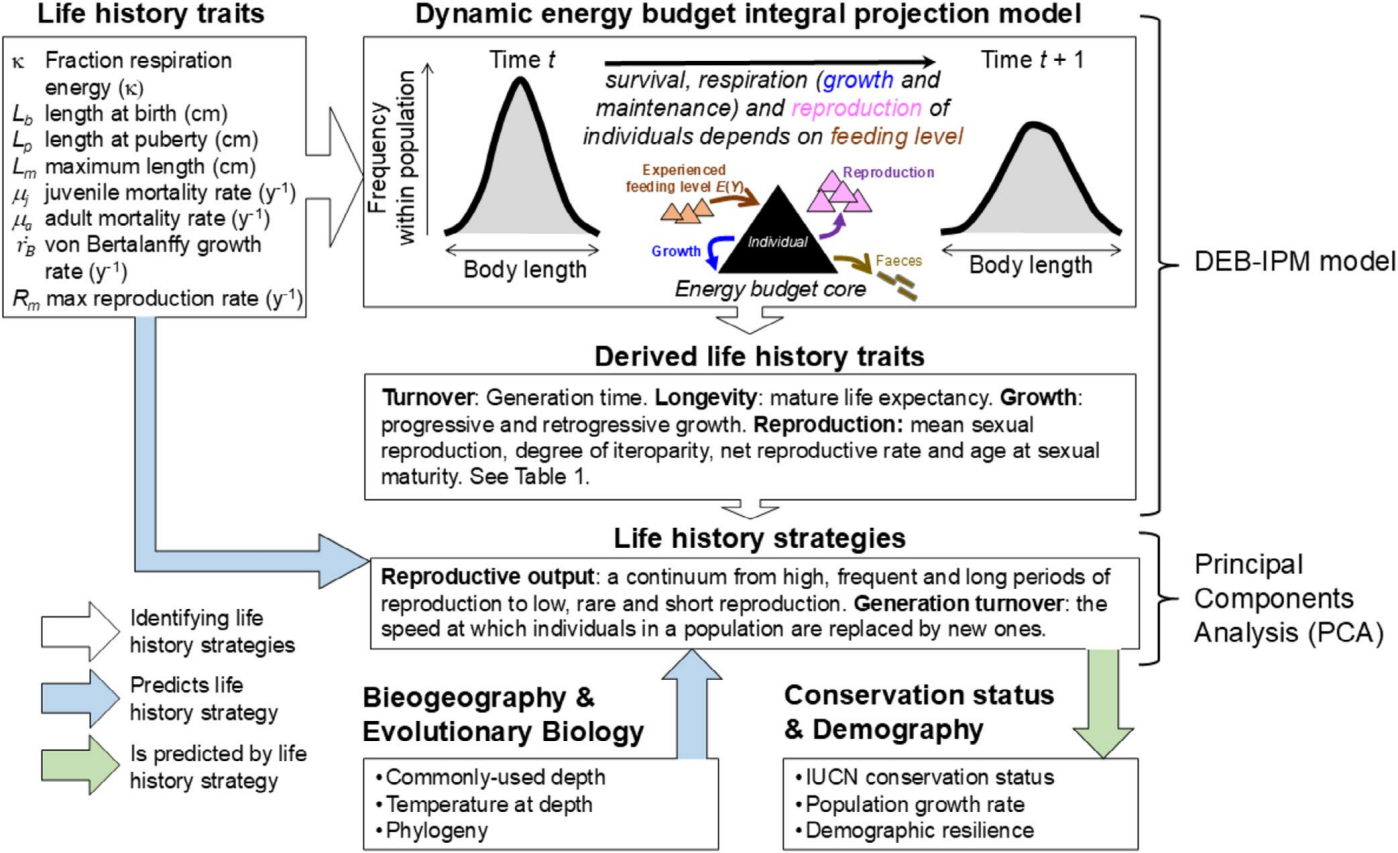
Workflow of parameterising a DEB‐IPM using the DEBBIES database (Smallegange and Lucas 2024), using the derived life history traits to plot elasmobranch life history strategies in a Principal Components Analysis (PCA). Species biogeography and phylogeny were used to estimate position on PCA axes, and the PC scores were used to predict conservation status, population growth rates and demographic resilience. White arrows indicate parameterisation of models or calculation of traits. Blue arrows represent metrics used to predict life history strategies and green arrows indicate the use of life history strategies to make predictions of conservation status and demography. Adapted from Smallegange and Lucas (2024).

A DEB‐IPM describes the dynamics of a population comprising cohorts of females of different lengths that survive, grow and reproduce (Smallegange et al. 2017; Smallegange and Lucas 2024). A DEB‐IPM has eight parameters (Figure 1: top‐left box), which we initially parameterised for 157 elasmobranch species, from 11 orders and 37 families, using the DEBBIES database (Smallegange and Lucas 2024; downloadable at Smallegange 2020).

In brief, the demographic functions that describe growth and reproduction in a DEB‐IPM are derived from the Kooijman‐Metz model (Kooijman and Metz 1984) (see Appendix, section 1 in Figshare repository: Lucas et al. 2024), which is a simple version of the standard model of Kooijman's DEB theory and only includes the tradeoff between growth and reproduction, but still fulfils the criteria for general explanatory models for the energetics of individuals (Sousa et al. 2010). The Kooijman‐Metz model assumes that organisms are isomorphic, are born at length *L*_b_, mature at length *L*_p_ and ingest food at a rate that is proportional to their experienced feeding level *E*(*Y*). This experienced feeding level reflects food availability according to observed gut fullness for fish, ranging from completely empty (0 ≤ *E*(*Y*) < 0.1), to very few food particles (0.1 ≤ *E*(*Y*) < 0.3), ‘contents divided in different patches’ (0.3 ≤ *E*(*Y*) < 0.7), ‘just filled’ (0.7 ≤ *E*(*Y*) < 0.9), or ‘completely full’ (0.9 ≤ *E*(*Y*) ≤ 1) (Piet and Guruge 1997). For example, in reef manta rays (*Mobula alfredi*) a feeding level of *E*(*Y*) = 0.8 is associated with natural, stable populations (Smallegange et al. 2017). At the highest feeding level of *E*(*Y*) = 1, individuals grow to a maximum length *L*_m_. As individuals feed and uptake energy, a constant fraction κ of assimilated energy is allocated to respiration to cover maintenance costs and somatic growth. The remaining fraction 1—κ of assimilated energy is allocated to reproduction in the case of adults and to the development of reproductive organs in the case of juveniles. Note that this role of κ in mediating the trade‐off between growth versus reproduction is implicit: *R*_m_ is a composite parameter that includes (1 − *κ*) whereas *L*_m_ is a composite parameter that includes κ (Kooijman and Metz 1984). Importantly, both *R*_m_ and *L*_m_ are empirically derived from data rather than computed directly from their constituent components. The parameter κ only enters a DEB‐IPM explicitly to define the starvation condition, where individuals of length *L* die when $L>L_{m}\cdot E\left(Y\right)/\kappa$ (see further Appendix, section 1 in Figshare repository: Lucas et al. 2024). Here, κ functions as a direct input parameter (Figure 1), influencing survival thresholds rather than being inferred from composite traits. If an individual survives from year *t* to year *t* + 1 (juveniles have a different mortality rate *μ*_j_, than adults, *μ*_a_) it grows in length following a von Bertalanffy growth curve characterised by ${\dot{r}}_{B}$; the von Bertalanffy growth rate. If a surviving female is an adult, she also produces offspring at a maximum rate of *R*_m_ if she experiences the highest feeding level of *E*(*Y*) = 1.

### 2.2 Life History Strategies

Eight representative life history traits were calculated based on schedules of survival, growth and reproduction (Table 1; Salguero‐Gómez et al. 2016; Salguero‐Gómez 2017; Capdevila, Beger, et al. 2020) in MATLAB Version 9.12 (The MathWorks Inc. 2022) using the code in Smallegange and Lucas 2024 (objective 1). For each species, this was done for a low and high feeding level, *E*(*Y*) = 0.6 and *E*(*Y*) = 0.9, respectively. *E*(*Y*) = 0.6 reflects unfavourable feeding conditions, whereas a feeding level of *E*(*Y*) = 0.9 represents a full gut and thus favourable food availability (Piet and Guruge 1997). Feeding levels lower than *E*(*Y*) = 0.6 were excluded because these were too low for individual growth and reproduction to occur for many species. Specifically, at the length where $L>L_{m}E\left(Y\right)$, individuals shrink and might not reach their maturation size (*L*_p_). Furthermore, individuals die of starvation at length $L>L_{m}E\left(Y\right)/\kappa$ (Appendix, section 1 in Figshare repository: Lucas et al. 2024). If *E*(*Y*) = κ, then individuals die of starvation at their maximum length. For most species, we set κ = 0.8 (Smallegange 2020). Thus, for *E*(*Y*) values lower than 0.8, many individuals smaller than the maximum length may die of starvation. These factors combined mean that as feeding level drops, more species experience declining population growth rate and extinction. To assess if this reduction in species number was biased by species' life history traits, we applied a linear discriminant analysis (LDA) on the scaled DEB‐IPM input parameters. We assigned two prior groupings: species that persist at the low feeding level (*n* = 63) and species that persist only at the high feeding level (*n* = 88). The LDA model was fitted using the *lda* function from the MASS package (Venables and Ripley 2002) in R (R Core Team 2023) and captured all variation with one discriminant function (LD1). LD1 had high Wilks' lambda values (λ_W_ = 0.99), indicating weak separation, with a non‐significant associated discriminant function (LD1: *F*_1,149_ = 1.19; *p* = 0.278). This confirms that there was no bias in species composition in the final dataset for analysis.

**TABLE 1:** Demographic quantities and their loadings onto the Principal Components axes.

PCA type				pPCA			Mass‐corrected pPCA		
Number of populations in analysis				214 (151 species)			168 (117 species)		
Demographic quantity	Symbol	Definition	Equation	p‐PC1	p‐PC2	p‐PC3	p‐PC1	p‐PC2	p‐PC3
Generation time	*T*	Number of years required for the individuals of a population to be fully replaced by new ones	$T=\frac{\log\left(R_{0}\right)}{\log\left(\lambda \right)}$	**0.912**	−0.149	0.295	**0.517**	**−0.774**	0.300
Age at maturity	*L*_α_	Number of years that it takes an average individual in the population to become reproductive	$L_{\alpha }=\eta_{L_{b}}$	**0.814**	0.353	0.048	−0.064	−0.203	0.949
Progressive growth	*γ*	Mean probability of growing to a larger length across the length domain Ω.	${\left.\gamma =\sum_{i}^{m}{\bar{G}}_{i,j}\right|}_{i<j}$	**0.877**	0.039	0.029	−0.083	**−0.945**	0.234
Retrogressive growth	*ρ*	Mean probability of growing to a smaller length across the length domain Ω.	${\left.\rho =\sum_{i}^{m}{\bar{G}}_{i,j}\right|}_{i>j}$	−0.128	**−0.890**	0.196	**0.889**	0.173	−0.223
Mean recruitment success	*φ*	Mean per‐capita number of recruits across the length domain Ω.	$\varphi =\sum_{i}^{m}{\bar{V}}_{i,j}$	**−0.573**	**0.699**	−0.334	**−0.921**	0.283	−0.008
Degree of iteroparity	*S*	Coefficient of variation in age at reproduction	$S={\bar{m}}_{x}/\sigma \left(m_{x}\right)$	−0.068	0.250	−0.963	**−0.888**	0.062	0.086
Net reproductive rate	*R*_0_	Mean number of recruits produced during the mean life expectancy of an individual in the population.	$R_{0}=\sum_{x=0}^{x=\eta_{e}}l_{x}m_{x}$	0.288	**0.936**	−0.096	**−0.797**	−0.054	0.546
Mature life expectancy	*L*_ω_	Number of years from the mean age at maturity (*L*_α_) until the mean life expectancy (*η*_e_) of an individual in the population.	$L_{\varpi }=\eta_{L_{p}}$	**0.914**	0.208	−0.135	−0.312	−0.464	0.754
Axis				**Turnover**	**Repro**	—	**Repro**	**Turnover**	—
Proportion of variance explained				45.5%	34.8%	8.8%	48.3%	34.9%	7.8%
Cumulative proportion of variance explained				45.5%	80.3%	89.1%	48.3%	83.2%	91.0%
Kaiser criterion				13.28	7.76	0.50	14.9	7.79	0.39
Pagel's lambda (for the pPCA)				0.417			0.335		

*Note:* To calculate life history traits, we discretised each DEB‐IPM (Equation 1) by dividing the length domain Ω into 200 very small‐width discrete bins, resulting in a matrix **A** of size *m* × *n*, where *m* = *n* = 200, and which dominant eigenvalue equals *λ*. Mean lifetime reproductive success *R*_0_ is the dominant eigenvalue of the matrix $F=V{\left(I-\mathrm{GS}\right)}^{-1}$, where **I** is the identity matrix and **V** = **DR**, with **D** as the parent‐offspring association, **R** the reproduction, **G** the growth and **S** the survival matrix (Caswell 2001); this gives generation time *T* = log(*R*_0_)/log(*λ*) (Caswell 2001). The mean life expectancy, *η*_e_, is calculated as *η*_e_ = 1^T^ **N**, where 1 is a vector of ones of length *m* and **N** is the fundamental matrix $N={\left(I-S\right)}^{-1}$. The longevity of an individual of length *L* is *η*_L_, which means we can calculate age at sexual maturity $L_{\alpha }=\eta_{L_{b}}$ and mature life expectancy $L_{\varpi }=\eta_{L_{p}}$ so that $\eta_{e}=L_{\alpha }+L_{\varpi }$ (Caswell 2019, equation 4.21). *l*_x_ is the probability of surviving to age at least *x*, and *m*_x_ is the average fertility of age class *x* (cf. Tuljapurkar et al. 2009), $\bar{G}$ is the mean of G, $\bar{V}$ is the mean of V, and *i* and *j* are the row and column entries of the matrix, respectively. The vital rates included in the studied set of demographic quantities (progressive growth *γ*, retrogressive growth *ρ*, and sexual reproduction *φ*) were averaged across the columns *j* (the length bins), weighted by the relative contributions of each stage at stationary equilibrium. For example, to calculate mean sexual reproduction *φ*, we summed the values in the columns *j* of the **V** matrix and multiplied each *φ*_*ij*_ by the corresponding *j*th element *w*_j_ of the stable stage distribution *w*, calculated as the right eigenvector of **A**. Only loadings for the pPCA and mass‐corrected PCA are reported, as Pagel's λ_P_ > 0.25. Kaiser criterion > 1 only for PC1 and PC2, so PC3s were not retained. Bold values indicate strong loadings on principal components 1 and 2.

To identify life history strategies along major axes (objective 2), we performed a varimax‐rotated, phylogenetically corrected principal component analysis (pPCA) (Revell 2012), using the *phy.pca* function in the R library *phytools* (Revell 2012; R Core Team 2023). The phylogenetic correction was conducted at the species level, where for each of the 63 species that were present in both the low and high feeding levels, each duplicate was treated as a separate population of the same species using a dichotomous branch at the species' tip (Paniw et al. 2018; Healy et al. 2019). Traits were log‐transformed and scaled to adhere to PCA assumptions (μ = 0 and SD = 1). We assessed the significance of PC axes using Kaiser's criterion, retaining PC axes with eigenvalues greater than unity (Kaiser 1960).

We report results from one single phylogenetic tree (see Appendix, Figure S1 in Figshare repository: Lucas et al. 2024), randomly selected from a subsample of 20 possible tree configurations from Stein et al. (2018), available at Vertlife.org. For an explanation of tree selection, see Appendix, section 3 and File S2 in Figshare repository: Lucas et al. (2024). Six species were excluded from the analyses as they were either not in the Vertlife.org database (*Squalus hawaiiensis*, *Aetobatus narutobiei* and *Maculabatis ambigua*), they did not have an associated polygon shapefile (see below) on the IUCN database ( *Etmopterus granulosus* and *Aetomylaeus bovinus*), or were identified as an extreme outlier after visual inspection of the PCA due to extreme life history trait values compared to other species in the dataset ( *Somniosus microcephalus* ). Therefore, the analysis was conducted on 151 species.

We checked the influence of phylogeny and body mass on the structuring of life history strategies (see Jeschke and Kokko 2009 for a detailed discussion) by running PCA analyses with and without a phylogenetic correction, and with and without a body mass correction. The pPCA links the phylogeny to the life‐history traits via a modified covariance matrix and estimated Pagel's λ_P_, a scaling parameter for the phylogenetic correlation between species, expected under Brownian motion (Freckleton et al. 2002). Pagel's λ_P_ was estimated using the *ape* package (Paradis and Schliep 2019) and varies between 0 (phylogenetic independence) and 1 (species traits covary proportionally to their shared evolutionary history) (Revell 2010).

Body mass was corrected for by computing the residuals for linear models between the log_10_‐transformed life history traits (Table 1) and body mass for each species for both the PCA and the pPCA (Jolicoeur et al. 1984; Gaillard et al. 1989; Revell 2009). Body mass values were extracted from Fishbase (Froese and Pauly 2023) using the *rfishbase* package (Boettiger et al. 2012). Where available, we used the maximum mass given on Fishbase. Otherwise, we employed length‐weight relationships to infer maximum body mass. If the length‐weight relationship used a length type that was not in the database, length‐length conversions were conducted prior to length‐weight conversions. Data were not available for 34 species, so there were 151 species in the main analyses and 117 species in the analyses containing body mass corrections (Table 1).

Pagel's λ_P_ values exceeded the threshold of 0.25 in each analysis, indicating a non‐negligible phylogenetic signal and justifying the use of pPCA. Comparisons of trait loadings between uncorrected and mass‐corrected analyses revealed qualitative differences, with traits loading onto different principal components. The difference in trait covariation was driven by the inclusion of body mass correction, rather than by a change in species composition between the two datasets (see Appendix, Table S2 in Figshare repository: Lucas et al. 2024). This suggests that both phylogeny and body mass exerted a structuring influence on trait covariation, potentially obscuring biologically meaningful axes of life history variation. The combined use of phylogenetic and mass corrections thus provides a more robust framework for identifying evolutionary patterns in life history traits.

Finally, to compare if and how species position in the life history space changed between the low and high feeding levels, we conducted Pearson's product–moment correlation tests between feeding level (*E*(*Y*)) and PC scores for each axis.

### 2.3 Effect of Clade and Habitat (Water Temperature) on Life History Patterns

We assessed if habitat and clade affect elasmobranch life history variation (objective 3). We sourced habitat information (water depth and temperature) for each species. Continuous variable ‘commonly used depth’ was sourced for each species from the Fishbase database (Boettiger et al. 2012; Froese and Pauly 2023). If commonly used depth was unavailable, median depth was sourced from the IUCN database (IUCN 2024). For temperature, we used ‘temperature‐at‐depth’ following the methodology of (Barrowclift et al. 2023). To this end, shape files containing polygons of known depth ranges for each species were taken from the IUCN database (IUCN 2024). These were overlaid onto the International Pacific Research Center's mean annual ocean temperatures across 27 depth levels (0–2000 m), based on a dataset from the Argo Project (available at http://apdrc.soest.hawaii.edu/projects/Argo/data/statistics/On_standard_levels/Ensemble_mean/1x1/m00/index.html). Median temperature‐at‐depth was calculated using values at the nearest depth level to each species' commonly used depth (or the IUCN median depth where commonly used depth was unavailable). For regionally endothermic species (family *Lamnidae*, Dickson and Graham 2004) a correction factor of 3.5°C was applied to their median temperature, consistent with a similar demographic study by Pardo and Dulvy (2022).

We tested for collinearity between depth, temperature and clade using visual inflation factor (threshold value VIF > 10) in the *faraway* R package (Faraway 2022). Depth, temperature and clade were all collinear. Therefore, we separately assessed the effect of temperature at depth (as the representative variable for habitat) and clade on the PC scores of the first and second axis of the (p)PCA. We used two general linear mixed‐effect models (LMERs) where the predictor variable was either temperature at depth or clade, with a random effect of species in each model, and the response variable was either PC1 or PC2 score. For each LMER, the model assumptions of Gaussian errors and homoscedasticity were confirmed by inspecting the probability plots and error structures in R (Lüdecke et al. 2021; Faraway 2022; R Core Team 2023).

### 2.4 Life History Strategies as Predictors of Population Growth Rate, Demographic Resilience and Conservation Status

We used linear models (LMs) to test if the PC scores (continuous variables) of the first and second axis of the best fitting PCA, and their interaction, predict population growth rate (λ) and demographic resilience across species (objective 4). Even though PCA assumes that the principal components (PC1 and PC2) are uncorrelated, this assumption pertains to the original data's variance structure. By testing interactions between PC1 and PC2 we explored whether the combined influence of these principal components provides additional explanatory power for our response variable, beyond their individual effects. To calculate λ, we discretised each DEB‐IPM (Equation 1) by dividing the length domain Ω into 200 very small‐width discrete bins, resulting in a matrix **A** of size *m* × *n*, where *m* = *n* = 200, and which dominant eigenvalue equals λ (Table 1). Demographic resilience was calculated as the damping ratio *ξ*, with $\xi =\lambda /|\left|\lambda_{2}\right||$, where λ_2_ is the highest subdominant eigenvalue of matrix **A** (Caswell 2001; Capdevila, Stott, et al. 2020). Alternative metrics exist to capture resilience (simulated time‐to‐recovery or transient growth rates [Capdevila et al. 2021]) but for ease of comparison with previous studies we used the damping ratio. To investigate predictive links between life history strategies and conservation status, global conservation statuses for each species were sourced from the IUCN database (IUCN 2024), where LC indicates Least Concern, NT indicates Near Threatened, VU indicates Vulnerable, EN indicates Endangered, CR indicates Critically Endangered and DD indicates Data Deficient. We used an ordinal regression to assess if PC scores predict species IUCN conservation status (excluding Data Deficient species).

All LMs, LMERs, ordinal regression analyses and plots (Wickham 2016; Attali and Baker 2023; Hijmans 2023; Slowikowski 2023) were performed in R version 4.3.2 (R Core Team 2023) in Rstudio (RStudio Team 2023), using the *dplyr* and *tibble* packages for data manipulation (Wickham et al. 2022; Müller and Wickham 2023). For each LM, the model assumptions of Gaussian errors, homoscedasticity and collinearity were confirmed by inspecting the probability plots and error structures in R (Lüdecke et al. 2021; Faraway 2022; R Core Team 2023).

## 3 Results

### 3.1 Two Axes Structure Elasmobranch Life History Strategies (Objective 1 and 2)

**FIGURE 2:**
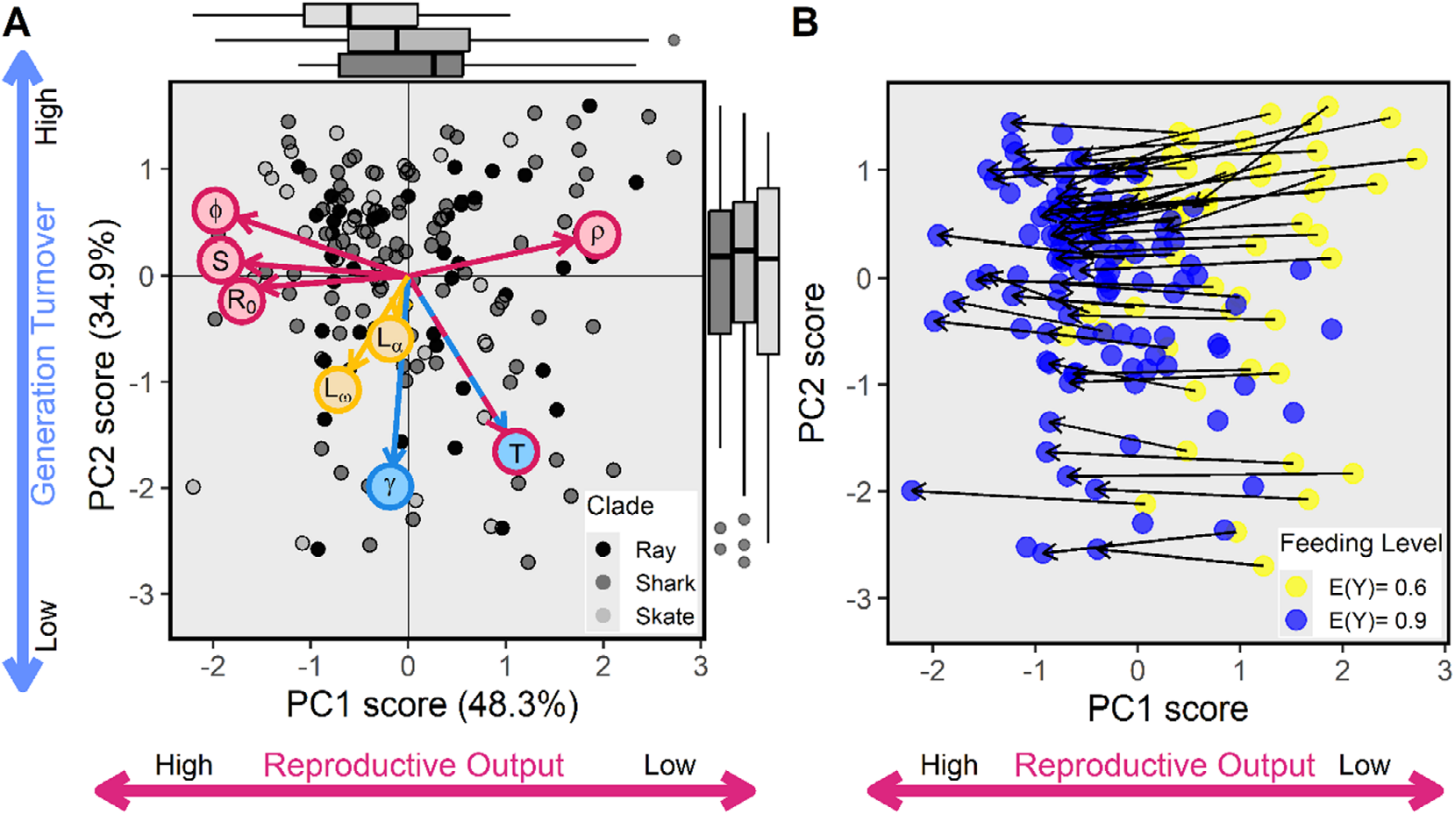
Mass‐corrected (p) PCA plots including 117 species from the high feeding level model (E(Y) = 0.9) and 51 of these species from the low feeding level model (E(Y) = 0.6). The 51 species that were in both high and low feeding level models were treated as independent populations, leading to a total of 168 data points in each plot (A) and (B). (A) Marginal boxplots of the split by clade (Shark, Skate and Ray) are included for each principal component. Labelled arrows correspond to the variables used in the PCA analysis and show the magnitude and direction of the loadings. Magenta arrows align with the reproductive axis, blue arrows align with the generation turnover axis and yellow arrows are not aligned with either PC1 or PC2. The dashed blue/magenta arrow aligns with both the reproductive output and generation turnover axes. (B) The change in PC scores is marked by arrows for 51 species in both low (yellow) and high (blue points) feeding level. 66 species appear only in blue, with no connected arrow. The change in life history strategies between low and high feeding level creates a shift towards higher reproductive output.

We found that two separate PC axes structure elasmobranch life history traits, cumulatively explaining 83.2% of the total variance in life histories (Table 1: PC1: 48.3%, PC2: 34.9%). Pagel's λ_P_ was higher than our cut‐off value of 0.25 (Pagel's λ_P_ = 0.31), indicating the phylogenetic signal was significant. In addition, correcting for body mass changed the dominant axes (Table 1) and we thus continued with the mass‐corrected pPCA. Degree of iteroparity (*S*), mean recruitment success (*φ*) and net reproductive rate (*R*_*0*_) were loaded negatively onto PC1, whereas retrogressive growth (*ρ*) and generation time (T) were loaded positively (Figure 2A and Table 1). These life history traits are all associated with reproductive output. Thus, as PC1 scores move from positive to negative, elasmobranchs attain greater lifetime reproductive success while their retrogressive growth and generation times decrease (Figure 2A). Generation time (*T*) and progressive growth (*γ*) were loaded negatively onto PC2 (Figure 2A and Table 1). As PC2 scores move from positive to negative, individuals increase their allocation to growth while population turnover decreases (i.e., greater generation time), so this axis is described as ‘generation turnover’ (Figure 2A). Finally, mean age at maturity (*L*_*α*_) and mature life expectancy (*L*_*ω*_) were not loaded onto either PC1 or PC2 (Table 1). The Kaiser criterion did not meet the threshold (< 1) for PC3, so this axis did not explain sufficient variance in life history strategies to be included in further analyses.

Feeding level was negatively correlated with PC1 score (reproductive output), *r*(166) = −0.639, *p* < 0.001. As feeding level increases, PC1 scores decrease, corresponding to an increase in lifetime reproductive output between the low and high feeding levels as species can spend more energy to reproduce at a higher rate (Figure 2B). There was no correlation between PC2 scores (generation turnover axis) and feeding level (*p* = 0.186).

### 3.2 Habitat Type Shapes Life History Strategies Differently for Different Feeding Levels and Clades (Objective 3)

**FIGURE 3:**
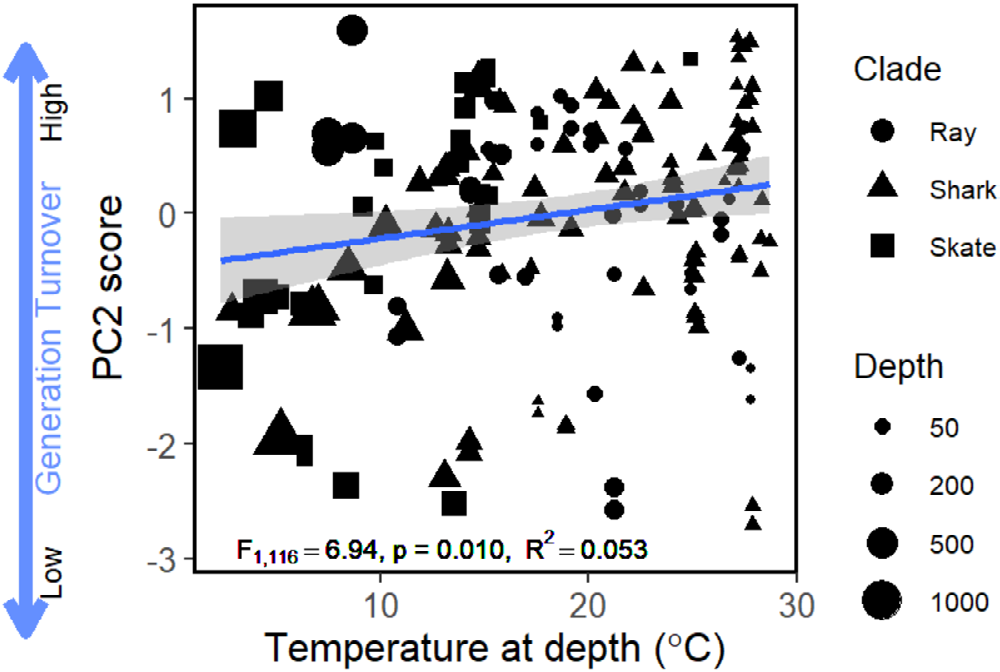
PC2 score decreased for increasing temperature‐at‐depth. Warmer temperatures relate to positive PC2 scores (higher generation turnover). The *F*‐statistic, *p*‐value and Marginal *R* ^2^ (variance explained by fixed effects only) are in the plot window. Conditional *R* ^2^ (variance explained by fixed and random effects) was 0.963. The grey band around the fitted models shows the 95% confidence interval.

Temperature did not significantly affect PC1 score (reproductive output) (*p* = 0.423). There was a significant effect of Clade on PC1 score (*F*_2,165_ = 4.12, Marginal *R* ^2^ = 0.047, *p* = 0.008). PC1 scores were lower for skates than for rays and sharks, indicating that skates have a high reproductive output (Figure 2A). PC2 scores (generation turnover) increased with increasing temperature at depth (*F*_1,116_ = 6.94, Marginal *R* ^2^ = 0.053, *p* = 0.010: Figure 3). This suggests that across all elasmobranchs, species with higher turnover occur in warmer waters. Phylogenetic clade did not significantly affect PC2 score (*p* = 0.762).

### 3.3 Life History Strategies Predict Population Performance, but Not IUCN Status (Objective 4)

**FIGURE 4:**
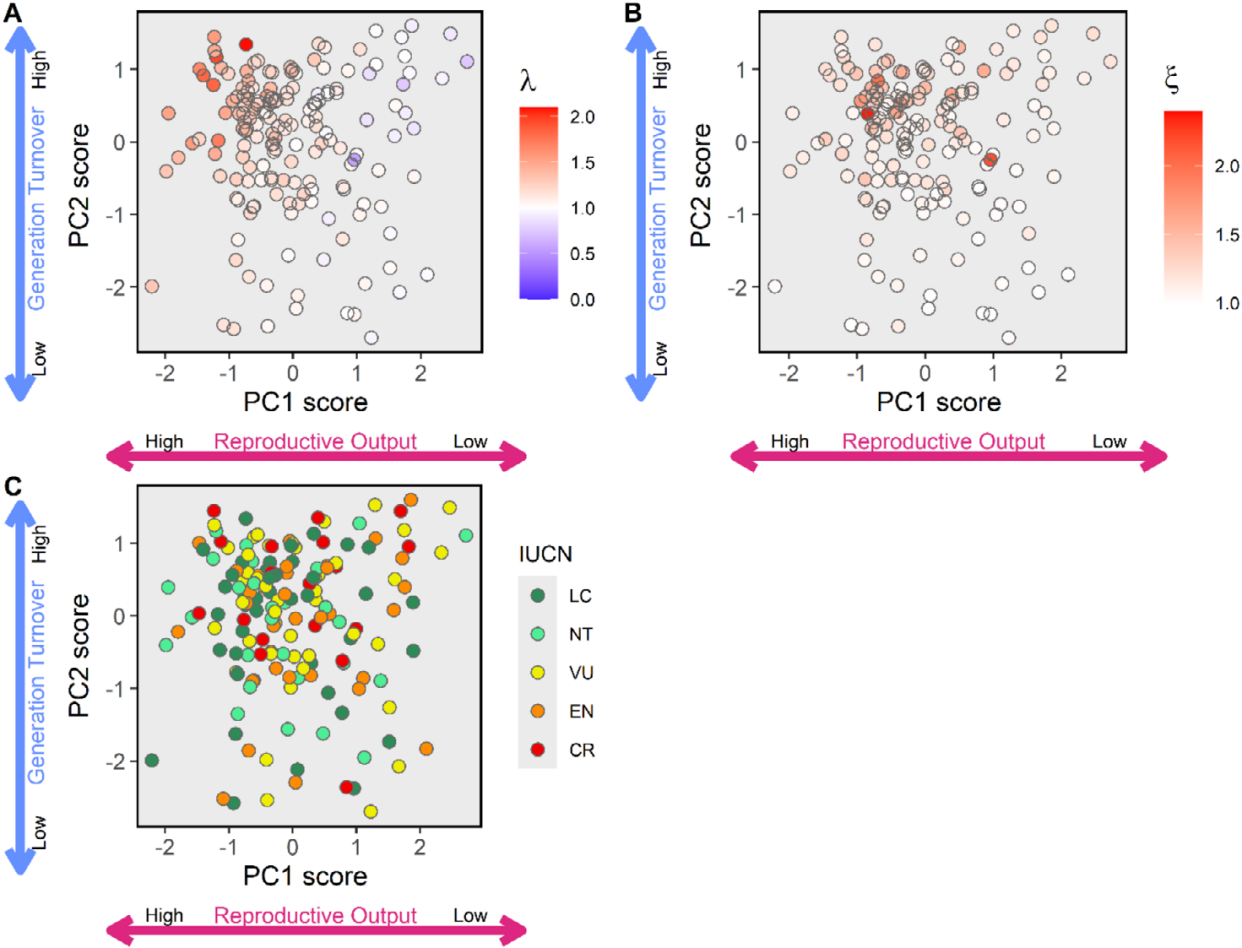
Overlays on the PCA figures for: Population growth rate (A), damping ratio (B), IUCN conservation status (C).

Population growth rate was significantly affected by the interaction between PC1 scores (reproductive output) and PC2 scores (generation turnover) (Table 2). Population growth rate increased with decreasing PC1 scores, but this increase was steeper for species with high, positive PC2 scores (higher turnover) than for species with high, negative PC2 scores (lower turnover) (Figure 4A). Thus, elasmobranch species with higher reproductive output and higher turnover experience higher population growth rates. Resilience to perturbations (damping ratio) significantly increased with decreasing PC1 scores (reproductive output) and increasing PC2 scores (generation turnover) (Table 2), showing that species with higher reproductive outputs and higher generation turnover have higher demographic resilience (Table 2 and Figure 4B). There was no significant association of PC1 scores, PC2 scores, or their interaction on the IUCN category of species (Table 2 and Figure 4C).

**TABLE 2:** General linear models for population growth rate (*λ*) and demographic resilience (damping ratio, *ξ*), as well as ordinal regression models for IUCN conservation status, as a response to PC1 (reproductive output) and PC2 (turnover) scores, for both the low (*E*(*Y*) = 0.6) and high (*E*(*Y*) = 0.9) feeding levels.

Variable	Axes	Estimate	df	Std. error	*p*	*R* ^2^
λ ~ PC1 × PC2	Intercept	1.173		0.010	**< 0.001**	0.619
	PC1	−0.154	1	0.011	**< 0.001**	
	PC2	0.062	1	0.010	**< 0.001**	
	PC1:PC2	−0.048	1	0.010	**< 0.001**	
	Error term	0.131	164			
*ξ* ~ PC1 × PC2	Intercept	1.199		0.014	**< 0.001**	0.167
	PC1	−0.037	1	0.015	**0.014**	
	PC2	0.081	1	0.015	**< 0.001**	
	PC1:PC2	−0.016	1	0.013	0.223	
	Error term	0.186	164			
as.factor (IUCN) ~ PC1 × PC2	PC1	0.231		0.143	0.107	0.007
	PC2	0.165		0.146	0.257	
	PC1:PC2	−0.019		0.129	0.881	

*Note:* *R* ^2^ values are adjusted‐*R* ^2^ for the linear models and pseudo‐*R* ^2^ for the ordinal regression on conservation status. McFadden's pseudo *R* ^2^ was calculated for the ordinal regression models using the equation $R^{2}=1-\frac{\text{residual deviance}}{\text{null deviance}}$. Bold values indicate statistically significant results (*p* < 0.05).

## 4 Discussion

Our aim was to investigate how individual energy budgets, phylogeny and habitat type structure elasmobranch life history variation, and if this structured variation can predict population performance and conservation biology. Elasmobranch life histories are captured by a reproductive output and generation turnover axis. Interestingly, we found that as feeding level increased, species moved towards higher reproductive output along the reproductive output axis but not the generation turnover axis. Regardless, the two life history axes predicted species' population growth rate and demographic resilience, but not IUCN conservation status.

### 4.1 The Impact of Feeding Level on Quantifying and Applying Life History Strategies

For many taxa, including elasmobranchs, their life history strategies are influenced by phylogeny and habitat (Capdevila, Beger, et al. 2020) as these impose selective pressures on the life history trajectories of individuals. In this study, we captured the phenomenon that species shift from lower to higher reproductive outputs as feeding level increases. In the core energy budget of our model, a fraction of the acquired energy is allocated to growth, while the remainder goes to reproduction (Kooijman and Metz 1984). This trade‐off is implicitly represented in our model through the composite parameters for maximum length (*L*_m_) and maximum reproduction rate (*R*_m_), which reflect the influence of κ and (1 − κ), respectively (Kooijman and Metz 1984). Although we use species‐specific estimates of κ as direct input for the starvation condition (see Appendix section 1), κ does not explicitly determine *L*_m_ or *R*_m_ in our implementation. As a result, while the trade‐off is structurally embedded in the model, we cannot directly assess how it is resolved across species based on our parameterisation. However, we can hypothesise how different resolutions of the growth‐reproduction trade‐off might influence species life histories as feeding levels increase. If a high proportion of energy is allocated to growth, an increase in feeding level would result in higher values of life history traits associated with growth (progressive growth *γ* and retrogressive growth *ρ*, respectively) (Smallegange and Guenther 2025). Conversely, if a high proportion of energy is allocated to reproduction, an increase in feeding level would enhance life history traits associated with reproduction (mean recruitment success *φ*, degree of iteroparity *S*, net reproductive rate *R*_0_ and mature life expectancy *L*_ω_) (Smallegange and Guenther 2025). These four traits together form our reproductive output axis.

If all elasmobranchs in our study allocate relatively more energy to reproduction than to growth, this would explain why changes in feeding levels are reflected solely along the reproductive output axis. Therefore, as shifts in feeding level drive the changes observed in our model, it is perhaps not surprising that the trait loadings in our analyses differ from previous studies, which typically identify the fast–slow continuum (pace‐of‐life axis) as the main dimension of life history variation across taxa (Capdevila, Beger, et al. 2020; Healy et al. 2019; Salguero‐Gómez 2017; Salguero‐Gómez et al. 2016). For example, in contrast to previous work (e.g., Salguero‐Gómez et al. 2016; Salguero‐Gómez 2017), we observed that progressive growth was higher in species with lower turnover rates, which we attribute to the wide range of von Bertalanffy growth rates in our dataset (Appendix section 4 and Figure S2, Lucas et al. 2024, Figshare repository).

In reality, species will vary in both energy acquisition and allocation as environmental conditions change. Understanding how these factors interact is key to predicting life history variation (Laskowski et al. 2021). For example, a pace of life axis can emerge if species differ in energy allocation at constant acquisition (feeding level), or if they differ in acquisition but have the same, constant allocation (Smallegange and Guenther 2025). However, individuals can also adjust energy allocation during their lifetime, redistributing it for maintaining functional stability in poor conditions or accelerating growth and reproduction when resources are abundant (Weidner et al. 2020). As a result, the same individuals within a population can show plasticity and change from a slow to fast pace of life, or vice versa, as energy availability varies over time (Del Giudice 2014). While within‐population links between life history and behaviour (pace of life syndrome; Dammhahn et al. 2018) are increasingly documented, their transferability across populations and species remains uncertain. For instance, Careau et al. (2009) observed a positive correlation between age at first reproduction and exploratory behaviour across 19 muroid species, with subsequent studies confirming similar within‐population patterns in house mice (Prabh et al. 2023) and common voles (Eccard et al. 2022). These findings suggest that while plasticity in life history and behaviour is evident within populations, its generalisability across taxa requires further investigation (Smallegange and Guenther 2025).

Plasticity in life history strategies can be adaptive when developing organisms adjust their allocation decisions in response to their local (feeding) environment, following evolved rules that maximise expected fitness in different ecological conditions (McNamara and Houston 1996; Ozgul et al. 2023). But the consequences of such plasticity and differences in the pace of life and other life history strategies across populations and species can be far‐reaching. Like others (Salguero‐Gómez et al. 2016; Salguero‐Gómez 2017), we found that two life history strategy axes predict species population growth and resilience. Perhaps unsurprisingly, both population growth and resilience increased with increasing feeding level along the reproductive output axis. However, a high population growth rate did not always equate to a high demographic resilience. For example, species with high reproductive output and generation turnover show high population growth rates but not always high resilience. In fact, complex environmental interactions and changes in pace of life can influence species extinction risk under climate change (Ozgul et al. 2023). However, our analysis did not find a link between life history strategies and IUCN threat status, likely because the IUCN status is fixed per species. Thus, plastic shifts in life history strategies would not predict threat status unless they impact it (as in Ozgul et al. 2023). All taken together, one should therefore be cautious when using life history strategies to predict how species perform in different environments.

### 4.2 Consequences for Elasmobranch Conservation

For many oceanic species, demographic data are sparce or lacking (Bradshaw et al. 2007; Pardo, Kindsvater, Cuevas‐Zimbrón, et al. 2016; Temple et al. 2020). This often means the only measure available to evaluate a species relative risk to perturbations like fishing or environmental change, is an estimate of its maximum intrinsic rate of population increase, *r*_max_ (Myers et al. 1997, 1999; Dulvy et al. 2004; Pardo, Kindsvater, Reynolds, and Dulvy 2016). Typically, species with higher *r*_max_ values are assumed to have higher resilience to perturbations (cf. Cortés 2016; Pardo, Kindsvater, Reynolds, and Dulvy 2016). The intrinsic rate of population increase, *r*, is related to population growth rate ($\lambda =e^{r}$). We found that species with the highest population growth rates do not necessarily show the highest resilience. This highlights the importance of exercising caution when using summary metrics such as *r*_*max*_ to predict species' resilience (cf. Smallegange et al. 2020). Our approach presents a more mechanistic methodology that can be applied to data‐sparce elasmobranchs to understand and predict their resilience and conservation status.

There is a broad diversity of diet and feeding strategies in elasmobranchs (Cortés et al. 2008), which potentially influence species‐specific life history strategy responses to changes in feeding levels. In this study, we found a consistent response between species to increase reproductive output with increased feeding level. However, when assessing individual species, researchers should consider realistic values and changes in feeding levels based on available literature or empirical studies to estimate population responses. Additionally, global (sea water) warming is linked to poorer hunting performance in sharks (Pistevos et al. 2015). Warming can thus reduce the sharks' experienced feeding level, population growth rate and ultimately their resilience. Understanding how elasmobranch life histories, their habitats and feeding translate into differences in resilience is crucial to informing management decisions and predicting conservation status. We did not find a relationship between life history axes and IUCN conservation status, possibly because plastic shifts in life history strategies were not linked to IUCN status (as discussed above), or because exploitation was not included in our models. Given that the most immediate threat to sharks, skates and rays is overfishing (Dulvy et al. 2021; Pacoureau et al. 2021; Worm et al. 2024), further study using an energy budget, demographic approach like ours could examine the effects of different fisheries mortalities (either by empirical measures or by derived estimates; Smith et al. 1998) on population performance of threatened or highly fished species.

## 5 Conclusion

Studies have shown that population responses to future environmental change and perturbations depend on species‐specific life‐history strategies (e.g., Ozgul et al. 2023). Further research should explore if variations in environmental conditions fuel plasticity of life‐history strategies in a range of taxa, and how these impact population performance and responses to change. Our analyses reveal that feeding level can cause plasticity in life‐history strategies, impacting how the dominant axes can be used to predict population responses to change and perturbations. For elasmobranchs, we provide strong support for the expansion of the classical use of maximum intrinsic rate of population increase, *r*_*max*_, to incorporating major axes of life‐history strategies, and highlight how our approach can be used to explore different scenarios of (over)fishing to quantify sustainable levels of exploitation.

## Author Contributions

**Sol Lucas:** conceptualisation, methodology, formal analysis, investigation, validation, data curation, writing – original draft preparation, writing – review and editing, visualisation; **Per Berggren:** writing – reviewing and editing, supervision; **Ellen Barrowclift:** methodology, formal analysis, writing – reviewing and editing; **Isabel M. Smallegange:** conceptualisation, methodology, formal analysis, investigation, validation, data curation, writing – original draft preparation, writing – review and editing, visualisation, supervision.

## Conflicts of Interest

The authors declare no conflicts of interest.

## Peer Review

The peer review history for this article is available at https://www.webofscience.com/api/gateway/wos/peer‐review/10.1111/ele.70201.

## Supporting information


**Data S1:** ele70201‐sup‐0001‐supinfo.docx.

## Data Availability

Data S1, including data and analysis code used to run the models in MATLAB and RStudio, are available at Figshare (DOI: https://doi.org/10.6084/m9.figshare.26166427). A publicly accessible pre‐print for this article is available at: https://doi.org/10.1101/2024.07.11.601909.
