# Supplementary material for: Changing Feeding Levels Reveal Plasticity in Elasmobranch Life History Strategies
Source: Ecol Lett. 2025 Sep 3;28(9):e70201. doi: 10.1111/ele.70201 (PMC12408948; doi:10.1111/ele.70201)
Supplement: Supplementary file 1 — Data S1: ele70201‐sup‐0001‐supinfo.docx. [file ELE-28-0-s001.docx]

**Appendix. Supporting Information to:**

**“Changing feeding levels reveal plasticity in elasmobranch life history strategies”**

Sol Lucas, Per Berggren, Ellen Barrowclift, Isabel M. Smallegange

*School of Natural and Environmental Sciences, Newcastle University,*

*Newcastle upon Tyne, NE1 7RU, UK*

**Contents:**

1. DEB-IPM equations
2. Principal component loadings check across twenty random phylogenetic trees
3. Pagel’s lambda and Kaiser criterion check across twenty random phylogenetic trees
4. Why was progressive growth higher for species with low turnover rates?
5. Supplementary figures and tables
6. References
7. **DEB-IPM equations**

The demographic functions that describe growth and reproduction in the DEB-IPM are derived from the Kooijman-Metz model^1^, which is a simple version of the standard model of Kooijman’s DEB theory, but still fulfils the criteria for general explanatory models for the energetics of individuals^2^. The Kooijman-Metz model assumes that individual organisms are isomorphic (body surface area and volume are proportional to squared and cubed length, respectively). The rate at which an individual ingests food, *I*, is assumed to be proportional to the maximum ingestion rate *I_max_*, the current feeding level *Y* and body surface area, and hence to the squared length of an organism: $I=I_{max}YL^{2}$. Ingested food is assimilated with a constant efficiency *ε*. A constant fraction *κ* of assimilated energy is allocated to respiration; this respiration energy equals $\kappa\varepsilon I_{max}YL^{2}$ and is used to first cover maintenance costs, which are proportional to body volume following $\xi L^{3}$ ($\xi$ is the proportionality constant relating maintenance energy requirements to cubed length), while the remainder is allocated to somatic growth. The remaining fraction 1 – *κ* of assimilated energy, the reproduction energy, is allocated to reproduction in case of adults and to the development of reproductive organs in case of juveniles, and equals $(1-\kappa)\varepsilon I_{max}YL^{2}$. This means that, if an individual survives from year *t* to year *t*+1, it grows from length *L* to length *L*’ following a von Bertalanffy growth curve, $\frac{dL}{dt}=\dot{r}_{B}\left( L_{m}\cdot Y-L \right)$, where $\dot{r}_{B}$ is the von Bertalanffy growth rate and $L_{m}=\kappa{\varepsilon I}_{max}/\xi$ is the maximum length under conditions of unlimited resource. Both *κ* and *I_max_* are assumed to be constant across experienced feeding levels, and therefore *L_m_* is also assumed constant. If a surviving female is an adult, she also produces offspring. According to the Kooijman-Metz model, reproduction, i.e. the number of offspring produced by an individual of length *L* between time *t* and *t* + 1, equals ${Y\cdot R}_{m}\cdot L^{2}/L_{m}^{2}$. The parameter *R_m_* is the maximum reproduction rate of an individual of maximum length *L_m_*. Note that *R_m_* is proportional to (1 – *κ*)^1^, whereas *L_m_* is proportional to *κ*, which controls energy conservation. However, the role of *κ* in the DEB-IPM is mostly implicit, as *κ* is used as input parameter only in the starvation condition (see below), whereas *R_m_* and *L_m_* are measured directly from data. Like *L_m_*, *R_m_* is also proportional to *I_max_*; since both *κ* and *I_max_* are assumed to be constant across experienced feeding levels, *R_m_* is also assumed constant.

The above individual life history events are captured in the DEB-IPM by four fundamental functions to describe the dynamics of a population comprising cohorts of females of different sizes^3^: (1) the survival function, $S\left( L(t) \right)$ (unit: y^-1^), describing the probability of surviving from year *t* to year *t*+1; (2) the growth function, $G\left( L^{'},L(t) \right)$ (unit: y^-1^), describing the probability that an individual of body length *L* at year *t* grows to length *L'* at *t* + 1, conditional on survival; (3) the reproduction function, $R\left( L\left( t \right) \right)$ (unit: # y^-1^), giving the number of offspring produced between year *t* and *t* + 1 by an individual of length *L* at year *t*; and (4) the parent-offspring function, $D(L^{'},L(t))$ (unit: y^-1^), the latter which describes the association between the body length of the parent *L* and offspring length *L*’ (i.e. to what extent does offspring size depend on parental size). The DEB-IPM assumes no effect of temperature on fundamental functions.

Denoting the number of females at year *t* by $N\left( L,t \right)$ gives the dynamics of the distribution of body length from year *t* to *t*+1 as:

$N\left( L^{'}, t+1 \right)=\int_{\Omega} \left[ D(L^{'},L(t))R\left( L\left( t \right) \right)+G{(L}^{'},L(t))S(L(t)) \right]N(L,t)dL$ eqn 1

where the closed interval Ω denotes the length domain. The survival function $S\left( L(t) \right)$ in equation (1) is the probability that an individual of length *L* survives from time *t* to *t* + 1:

$S(L(t))=\left\{ \begin{aligned} e^{-\mu_{j}} \mathrm{for} L_{b}\leq L<L_{p} {& L\leq L}_{m}E(Y)/\kappa& , \\ e^{-\mu_{a}} {\mathrm{for} L}_{p}\leq L\leq L_{m} {& L\leq L}_{m}E(Y)/\kappa\\ 0 o\mathrm{therwise} \end{aligned} \right.$, eqn 2

where *E*(*Y*) can range from zero (empty gut) to one (full gut), *L_b_* is length at birth, *L_p_* length at puberty and *L_m_* maximum attainable length (Fig. 1). Individuals die from starvation at body lengths at which maintenance requirements exceeds the total amount of assimilated energy, which occurs when $L>L_{m}\cdot E(Y)/\kappa$ and hence, $S\left( L\left( t \right) \right)=0$ (e.g. an individual of length *L_m_* will die of starvation if *E*(*Y*)< *κ*, where *κ* is the fraction of assimilated energy allocated to respiration, with 1 – *κ* allocated to reproduction [Fig. 1]). Juveniles and adults often have different mortality rates, and thus, juveniles ($L_{b}\leq L<L_{p}$) that do not die of starvation (i.e. $L\leq L_{m}\cdot E(Y)/\kappa$) have a mortality rate of μ_j_ and adults ($L_{p}\leq L\leq L_{m}$) that do not die of starvation (i.e. $L\leq L_{m}\cdot E(Y)/\kappa$) have a mortality rate of μ_a_ (Fig. 1).

In equation (1), the function $G\left( L^{'},L(t) \right)$ is the probability that an individual of body length *L* at time *t* grows to length *L'* at *t* + 1, conditional on survival, following a Gaussian distribution:

$G\left( L^{'},L(t) \right)=(\frac{1}{\sqrt{2\pi\sigma_{L}^{2}\left( L\left( t+1 \right) \right)}})e^{\frac{-{(L^{'}-E\left( L(t+1) \right)}^{2}}{2\sigma_{L}^{2}\left( L(t+1) \right)}}$ eqn 3

with the expected growth realised by a cohort of individuals with length *L*(*t*) equalling:

$E\left( L\left( t+1 \right) \right)=\left\{ \begin{aligned} L\left( t \right)e^{-\dot{r_{B}}}+{\left( 1-e^{-\dot{r_{B}}} \right)L}_{m}E(Y)\mathrm{for} L\leq L_{m}E(Y) \\ L(t) \mathrm{otherwise} \end{aligned} \right.$, eqn 4

and the variance in length at time *t* + 1 for a cohort of individuals of length *L* as:

$\sigma_{L}^{2}\left( L(t+1) \right)=\left\{ \begin{aligned} (1-e^{-\dot{r_{B}}})^{2}L_{m}^{2}\sigma^{2}\left( Y \right)\mathrm{for} L\leq L_{m}E(Y) \\ 0 \mathrm{otherwise} \end{aligned} \right.$ eqn 5

where *σ*(*Y*) is the standard deviation of the expected feeding level, and where $\dot{r}_{B}$ is the von Bertalanffy growth rate (Fig. 1).

The reproduction function $R\left( L(t) \right)$ in equation (1) gives the number of offspring produced between time *t* and *t* + 1 by an individual of length *L* at time *t*:

$R\left( L(t) \right)=\left\{ \begin{aligned} 0 \mathrm{for} L_{b}\leq L<L_{p} \\ {E\left( Y \right)R}_{m}{L\left( t \right)}^{2}/L_{m}^{2} &\mathrm{for} L_{p}\leq L\leq L_{m}E(Y) \\ \frac{R_{m}}{1-\kappa}\left[ E\left( Y \right){L\left( t \right)}^{2}-\frac{\kappa L(t)^{3}}{L_{m}} \right] &\mathrm{for} L_{m}E(Y)<L\leq L_{m}E(Y)/\kappa\end{aligned} \right.$eqn 6

Individuals are mature when they reach puberty at body length *L_p_* and only surviving adults reproduce; thus, only individuals within a cohort of length $L_{p}\leq L\leq L_{m}Y/\kappa$ reproduce.

Finally, the probability density function $D\left( L^{'},L(t) \right)$ gives the probability that offspring of an individual of body length *L* are of length *L'* at time *t* + 1, and hence describes the association between parent and offspring character values:

$D\left( L^{'},L\left( t \right) \right)=\left\{ \begin{aligned} 0 \mathrm{for} L<L_{p} \\ &\frac{1}{\sqrt{2\pi\sigma_{L_{b}}^{2}\left( L\left( t \right) \right)}}e^{\frac{-{(L^{'}-E_{L_{b}}\left( L\left( t \right) \right)}^{2}}{2\sigma_{L_{b}}^{2}\left( L\left( t \right) \right)}}\mathrm{otherwise} \end{aligned} \right.$ eqn 7

where $E_{L_{b}}\left( L\left( t \right) \right)$ is the expected size of offspring produced by a cohort of individuals with length *L*(*t*), and $\sigma_{L_{b}}^{2}(L\left( t \right))$ the associated variance. For simplicity, $E_{L_{b}}\left( L\left( t \right) \right)$ is set to be constant and associated variance,$\sigma_{L_{b}}^{2}\left( L\left( t \right) \right)$ is assumed to be very small.

1. **Loadings check across twenty random phylogenetic trees**

Phylogenetic trees may have multiple possible arrangements, for different estimates of the time passed from the most recent common ancestor between species. For the species in our analysis, we extracted twenty random phylogenetic trees, for the possible combinations of relatedness from Vertlife.org^4^. We ran the phylogenetically-corrected PCA (pPCA) for each tree. In file S1 (Supplementary material), the traits are shown in column A, by ‘lm’ followed their symbol (e.g. ‘lmT’ for Generation time), where ‘lm’ denotes that the traits have been log-scaled and regressed against body mass in the body mass correction procedure. Columns B-D are the loadings on PC1, PC2 and PC3 respectively. Each set of ten rows (starting with row 2:11) corresponds to one analysis, where the first nine rows (starting with 2:10) indicate the life history trait loadings, followed by the tenth row (starting with row 11), which shows the percentage variance explained for each axis. We completed a qualitative check, comparing the loadings for each random tree. The values of the loadings had minor differences, but the dominant loadings on each axis were consistent. Therefore, we proceeded with one phylogenetic tree for our analyses, randomly chosen from the twenty trees.

1. **Pagel’s lambda and Kaiser criterion check across twenty random phylogenetic trees**

To correct for phylogenetic relatedness among species, a pool of 10,000 possible phylogenetic trees were obtained from Stein et al. (2018)^4^, available at Vertlife.org. These trees represented 500 root node ages combined with 20 scenarios for infilling taxon with no genetic data. We randomly selected twenty trees (as done by Barrowclift et al., 2023^5^) and ran the pPCA for each. Each rooted phylogenetic tree had its branch lengths scaled proportionally based on time of separation of clades and species, using the rotl package^6^. For each of the twenty random phylogenetic trees, we checked the value of Pagel’s *λ* (with a cut-off of *λ* > 0.25) to determine the strength of phylogenetic signal^7,8^ and the Kaiser criterion (eigenvalues > 1) to assess the number of principal components to keep^9^. In file S2 (Supplementary material), column A shows the analyses in the form pl_phylo_mass[.Y], where [.Y] is the analysis number (where [.Y] is blank in cell A2 and up to ‘.19’ in cell A21). Column B contains the Pagel’s *λ* for each analysis. Across all analyses, Pagel’s λ was consistently >0.25, so we used the pPCA. Columns C-F contain the eigenvalues corresponding to PC1, PC2, PC3 and PC4, respectively. Across all analyses, PC1 and PC2 were all >1, whereas PC3 and PC4 values were always <1. Therefore we retained PC1-PC2 for all analyses.

1. **Why was progressive growth higher for species with low turnover rates?**

In our analyses, progressive growth was higher for species with lower turnover rates, not higher ones^10,11^ (Table S2). We surmise that this is due to high variation in the von Bertalanffy growth rate between species, driving a positive correlation between generation time and progressive growth (cf. van Noordwijk & de Jong 1986^12^). For example, the values of progressive growth and generation time change with different values of von Bertalanffy growth rate and feeding level. The relationship between generation time and progressive growth for the reef manta ray, *Mobula alfredi,* can be plotted for a range of feeding levels and values of von Bertalanffy growth rate (Fig. S2). For the same von Bertalanffy growth rate value, generation time increases with decreasing feeding level and progressive growth decreases. At the same time, generation time decreases with increasing von Bertalanffy growth rate values. The result is that, at the same feeding level (black solid lines in Fig. S2), progressive growth increases with decreasing values of von Bertalanffy growth rate,and simultaneously, generation time increases. Because our dataset covers a wide range of von Bertalanffy growth rate values, this would explain why we found that the slower a species’ life history speed, the higher its apparent progressive growth. These findings highlight the need for careful interpretation of correlations between life history traits calculated from demographic datasets^10,11,13–15^ (our objective 1), and how these are used to predict population characteristics^12^.

1. **Supplementary Figures and Tables**
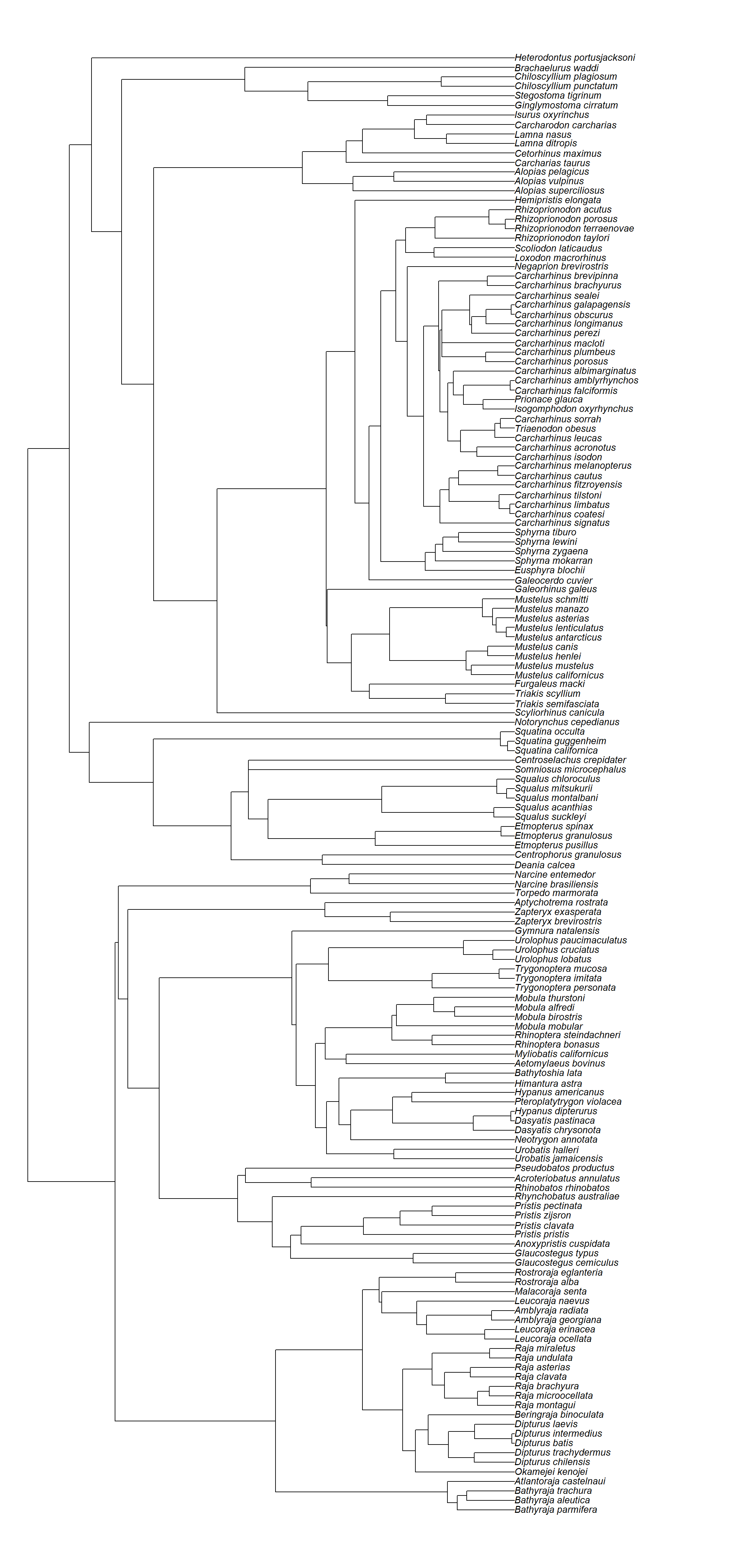


**Figure S1**. *Phylogenetic tree for 154 of the 157 species in the database. 3 species were not found from the database: S. hawaiiensis, A. narutobiei and M. ambigua*

| **Table S1.** Loadings and variance explained for the (p)PCAs with and without mass-correction, with eigenvalues >1. All species available were included in each analysis. Therefore, for mass-corrected models there were 168 populations, including 117 species and 51 species with populations for both the low (*E(Y)* = 0.6) and high (*E(Y)* = 0.9) feeding levels. For models without mass correction there were 214 populations, including 151 species and 63 species with populations for both the low and high feeding levels. Loading values greater than 0.5 are in bold. Pagel’s λ > 0.25 in each phylogenetic correction, so the pPCA was used. The qualitative check concluded that the loading of variables were different between the pPCA and the mass-corrected pPCA, so the mass-corrected pPCA was used in further analyses. | | | | | | | | | |
| --- | --- | --- | --- | --- | --- | --- | --- | --- | --- |
| **PCA** | **Full Model** | | **Mass-corrected** | | **Phylogenetically-corrected** | | **Phylogenetically and mass-corrected** | | |
| Eigenvalue >1 | PC1 | PC2 | PC1 | PC2 | PC1 | PC2 | PC1 | PC2 |  |
| Cumulative variance | 0.446 | 0.837 | 0.468 | 0.827 | 0.455 | 0.803 | 0.483 | 0.832 |  |
| Pagel’s lambda | - | | - | | 0.417 | | 0.335 | | |
| Number of populations | 214 (151 species) | | 168 (117 species) | | 214 (151 species) | | 168 (117 species) | | |
| *T* | 0.479 | 0.147 | 0.097 | **0.545** | **0.912** | -0.149 | **0.517** | **-0.774** |  |
| *L_α_* | 0.411 | -0.245 | **0.718** | -0.039 | **0.814** | 0.353 | -0.064 | -0.203 |  |
| *γ* | **0.507** | 0.055 | -0.083 | **0.737** | **0.877** | 0.039 | -0.083 | **-0.945** |  |
| *ρ* | -0.046 | **0.546** | -0.166 | -0.070 | -0.128 | **-0.890** | **0.889** | 0.173 |  |
| *φ* | -0.274 | -0.473 | 0.067 | -0.255 | **-0.573** | **0.699** | **-0.921** | 0.283 |  |
| *S* | 0.072 | 0.012 | -0.155 | 0.080 | -0.068 | 0.250 | **-0.888** | 0.062 |  |
| *R_0_* | 0.097 | **-0.621** | 0.481 | -0.117 | 0.288 | **0.936** | **-0.797** | -0.054 |  |
| *L_ω_* | **0.504** | -0.085 | 0.425 | 0.260 | **0.914** | 0.208 | -0.312 | -0.464 |  |

| **Table S2.** Comparison of trait loadings between uncorrected and mass-corrected phylogenetic principal component analyses (pPCA). The mass-corrected analysis included fewer species (117 species, 168 populations) than the uncorrected pPCA (151 species, 214 populations) due to missing body mass data. To isolate the effect of body mass from changes in species composition, we reran the uncorrected pPCA on the subset of species used in the mass-corrected analysis (117 species, 168 populations), without applying the mass correction. A qualitative comparison of trait loadings indicated that loadings from the subset pPCA were similar to those from the full uncorrected dataset. This suggests that differences in trait covariation were driven by the inclusion of body mass correction, rather than by a change in species composition between the two datasets. | | | | | | |
| --- | --- | --- | --- | --- | --- | --- |
| **PCA** | **Phylogenetically-corrected** | | **Phylogenetically and mass-corrected** | | **Phylogenetically corrected subset of species in the mass-corrected dataset** | |
| Eigenvalue >1 | PC1 | PC2 | PC1 | PC2 | PC1 | PC2 |
| Cumulative variance | 0.455 | 0.803 | 0.483 | 0.832 | 0.448 | 0.798 |
| Pagel’s lambda | 0.417 | | 0.335 | | 0.246 | |
| Number of populations | 214 (151 species) | | 168 (117 species) | | 168 (117 species) | |
| *T* | **0.912** | -0.149 | **0.517** | **-0.774** | **-0.907** | -0.161 |
| *L_α_* | **0.814** | 0.353 | -0.064 | -0.203 | **-0.807** | 0.344 |
| *γ* | **0.877** | 0.039 | -0.083 | **-0.945** | **-0.877** | 0.052 |
| *ρ* | -0.128 | **-0.89** | **0.889** | 0.173 | 0.144 | **-0.884** |
| *φ* | **-0.573** | **0.699** | **-0.921** | 0.283 | **0.554** | **0.718** |
| *S* | -0.068 | 0.25 | **-0.888** | 0.062 | 0.049 | 0.257 |
| *R_0_* | 0.288 | **0.936** | **-0.797** | -0.054 | -0.285 | **0.936** |
| *L_ω_* | **0.914** | 0.208 | -0.312 | -0.464 | **-0.913** | 0.182 |


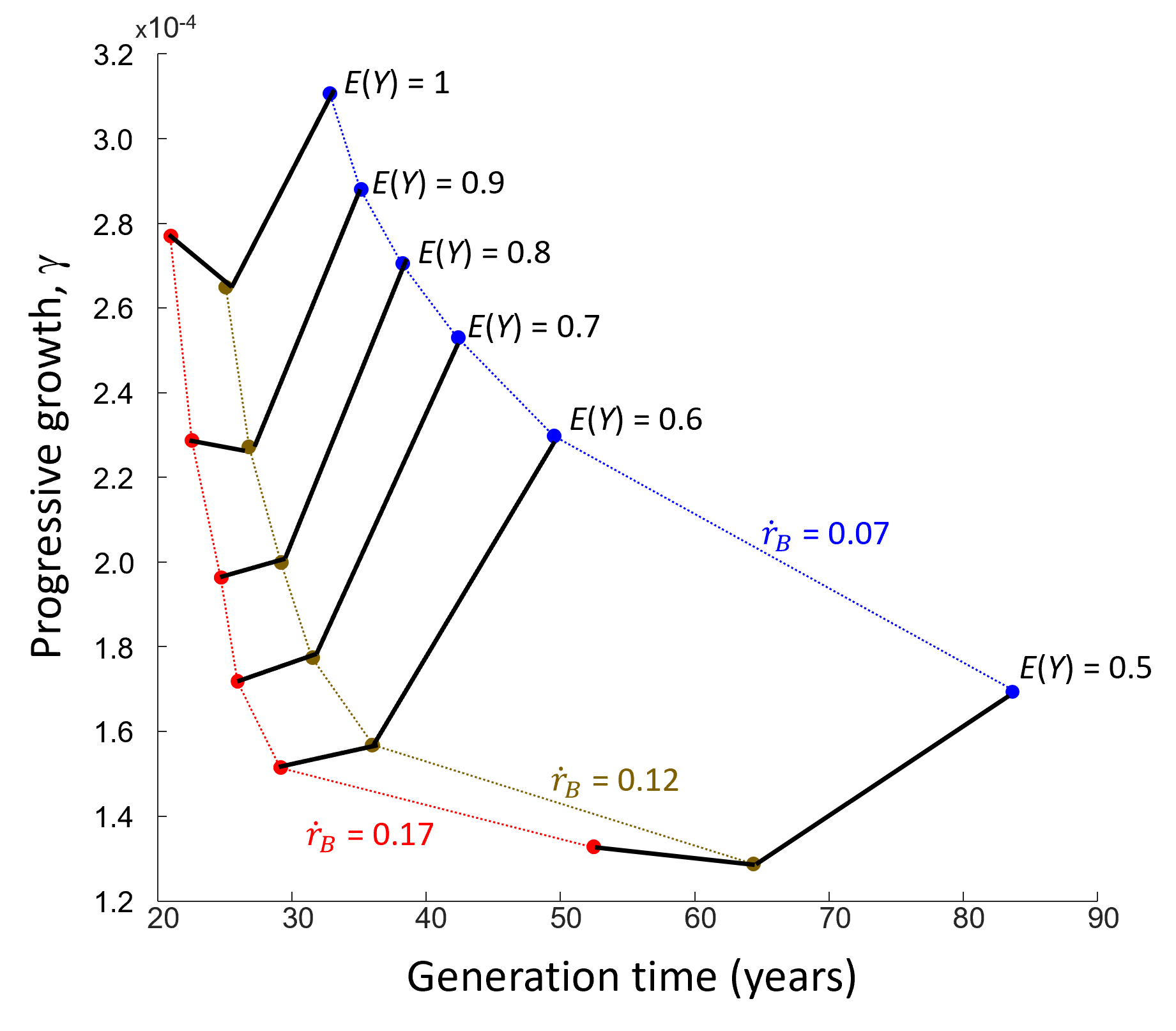


**Figure S2**. The relationship between generation time (years) and progressive growth, g, for the reef manta ray, Mobula alfredi in relation to feeding level E(Y) and the von Bertalanffy growth rate, $\dot{r}_{B}$. At the default value of $\dot{r}_{B}$=0.07 (Smallegange & Lucas, 2024)^16^ (blue dotted line), generation time increases with decreasing feeding level, which is what you would expect from life history theory, and progressive growth, γ, decreases. When we increase $\dot{r}_{B}$ to 0.12 (brown dotted line) and 0.17 (red dotted line) the same pattern exists, and, overall, generation time decreases and so does progressive growth. As a result, at almost each constant feeding level (black solid lines), progressive growth increases with decreasing $\dot{r}_{B},$and, at the same time, generation time increases. It thus appears that large variation in $\dot{r}_{B}$ within or between species and low variation in feeding level can drive a positive correlation between generation time and progressive growth (cf. van Noordwijk & de Jong 1986^12^). This would explain why we found that the slower a species pace of life, the higher is its progressive growth (Fig. 2) (and the lower its $\dot{r}_{B}$; see Results).

**6. References**

1. Kooijman, S. A. L. M. & Metz, J. A. J. On the dynamics of chemically stressed populations: The deduction of population consequences from effects on individuals. *Ecotoxicol Environ Saf* **8**, 254–274 (1984).

2. Sousa, T., Domingos, T., Poggiale, J. C. & Kooijman, S. A. L. M. Dynamic energy budget theory restores coherence in biology. *Philosophical Transactions of the Royal Society B: Biological Sciences* **365**, 3413–3428 (2010).

3. Smallegange, I. M., Caswell, H., Toorians, M. E. M. & de Roos, A. M. Mechanistic description of population dynamics using dynamic energy budget theory incorporated into integral projection models. *Methods Ecol Evol* **8**, 146–154 (2017).

4. Stein, R. W. *et al.* Global priorities for conserving the evolutionary history of sharks, rays and chimaeras. *Nat Ecol Evol* **2**, 288–298 (2018).

5. Barrowclift, E. *et al.* Tropical rays are intrinsically more sensitive to overfishing than the temperate skates. *Biol Conserv* **281**, 110003 (2023).

6. Michonneau, F., Brown, J. W. & Winter, D. J. rotl: an R package to interact with the Open Tree of Life data. *Methods Ecol Evol* **7**, 1476–1481 (2016).

7. Freckleton, R. P., Harvey, P. H. & Pagel, M. Phylogenetic analysis and comparative data: A test and review of evidence. *American Naturalist* **160**, 712–726 (2002).

8. Revell, L. J. Phylogenetic signal and linear regression on species data. *Methods Ecol Evol* **1**, 319–329 (2010).

9. Kaiser, H. F. The Application of Electronic Computers to Factor Analysis. *Educ Psychol Meas* **20**, 141–151 (1960).

10. Salguero-Gómez, R. *et al.* Fast–slow continuum and reproductive strategies structure plant life-history variation worldwide. *Proc Natl Acad Sci U S A* **113**, 230–235 (2016).

11. Salguero-Gómez, R. Applications of the fast–slow continuum and reproductive strategy framework of plant life histories. *New Phytologist* **213**, 1618–1624 (2017).

12. van Noordwijk, A. J. & de Jong, G. Acquisition and Allocation of Resources: Their Influence on Variation in Life History Tactics. *Am Nat* **128**, 137–142 (1986).

13. Paniw, M., Ozgul, A. & Salguero-Gómez, R. Interactive life-history traits predict sensitivity of plants and animals to temporal autocorrelation. *Ecol Lett* **21**, 275–286 (2018).

14. Healy, K., Ezard, T. H., Jones, O. R., Salguero-Gómez, R. & Buckley, Y. M. Animal life history is shaped by the pace of life and the distribution of age-specific mortality and reproduction. *Nat Ecol Evol* **3**, 1217–1224 (2019).

15. Capdevila, P. *et al.* Longevity, body dimension and reproductive mode drive differences in aquatic versus terrestrial life-history strategies. *Funct Ecol* **34**, 1613–1625 (2020).

16. Smallegange, I. M. & Lucas, S. DEBBIES Dataset to study Life Histories across Ectotherms. *Sci Data* **11**, 153 (2024).
